# A Transcriptomics Approach to Unveil the Antioxidant Effects of Tryptophan on Oocyte Quality Under Oxidative Stress in Pigs

**DOI:** 10.3390/biom15070949

**Published:** 2025-06-30

**Authors:** Zhekun Zhu, Yanlong Li, Xinyin Fan, Shuang Cai, Siyu Li, Yutian Wang, Xinyu Wang, Fengjuan Yang

**Affiliations:** 1 State Key Laboratory of Animal Nutrition and Feeding, College of Animal Science and Technology, China Agricultural University, Beijing 100193, China; b20213040362@cau.edu.cn (Z.Z.); s20223040732@cau.edu.cn (Y.L.); caishuang@cau.edu.cn (S.C.); bs20233040425@cau.edu.cn (S.L.); s20243040852@cau.edu.cn (Y.W.); b20213040352@cau.edu.cn (X.W.); 2 Beijing Key Laboratory of Bio feed Additives, China Agricultural University, Beijing 100193, China

**Keywords:** tryptophan, oxidative stress, oocyte quality, antioxidant enzymes, transcriptomics

## Abstract

This study investigates the effect of tryptophan treatment on aged pig oocytes, focusing on its potential to reduce oxidative stress and improve oocyte quality. An oxidative stress model was induced using hydrogen peroxide (H_2_O_2_) to mimic aging effects on oocytes. Fresh ovaries from young sows were collected, and oocytes were aspirated and cultured for in vitro maturation. Oocytes in the H_2_O_2_ and the H_2_O_2_+Trp groups were exposed to 100 µM H_2_O_2_ for 30 min, with the H_2_O_2_+Trp group receiving an additional 50 µM tryptophan supplementation. RNA-sequencing was performed to study the underlying mechanism through which tryptophan mitigated the H_2_O_2_-induced oxidative stress in oocytes. The results demonstrated that tryptophan supplementation significantly reduced oxidative stress markers such as H_2_O_2_ and malonaldehyde (MDA) while restoring key antioxidant enzymes such as superoxide dismutase (SOD), and catalase (CAT) confirming its antioxidant role. Furthermore, tryptophan improved cumulus cell expansion, and oocyte quality, which were compromised by oxidative stress. Transcriptomics study revealed the enrichment of several KEGG pathways, such as P13K-Akt signaling pathways as a critical regulator of cell survival and function, emphasizing the protective effects of tryptophan on oocyte integrity. Moreover, the protein–protein interaction (PPI) network identified several hub genes in the tryptophan-treated group compared with H_2_O_2_, including *TIMP1*, *CCN2*, and *MMP12* as key players in ECM remodeling and cellular adhesion, which are critical for restoring oocyte quality. These findings suggest that tryptophan supplementation not only mitigated oxidative stress but also modulated gene expression related to cellular functions and stress response. These results propose that tryptophan could be a valuable therapeutic strategy for improving reproductive outcomes in aging sows and other mammals facing age-related oocyte dysfunction.

## 1. Introduction

Reproductive success in mammals is intricately linked to the quality of oocytes, which can be significantly impacted by oxidative stress [1,2]. Oxidative stress is one of the major factors in the decline of oocyte quality, with reactive oxygen species (ROS) causing cellular damage that impairs oocyte viability and developmental potential [3,4]. Failure to fertilize or activate oocytes at the optimal time following ovulation can lead to a time-dependent decline in oocyte quality [2,5,6]. As the time since ovulation increases, ROS accumulates within the oocytes, potentially disrupting calcium balance and causing mitochondrial dysfunction [3,7]. While oocytes have a defense mechanism involving intracellular glutathione (GSH) to protect against oxidative damage [8], this protective system diminishes as the oocytes age post-ovulation [9,10]. The significant rise in ROS production, combined with the loss of antioxidant defenses, can lead to oxidative stress in oocytes. This stress may directly trigger apoptosis in oocytes that have aged after ovulation [11,12]. As such, improving oocyte quality by mitigating oxidative stress has emerged as a key goal in reproductive biology [13]. Therefore, minimizing oxidative stress in oocytes is crucial for enhancing oocyte quality and extending the window for oocyte-assisted reproduction.

Oocyte quality is crucial for successful reproduction, and its deterioration is often driven by oxidative stress, leading to structural and functional impairments such as DNA damage, reduced fertility, abnormal mitochondrial structures, and early oocyte apoptosis [3,14,15,16]. This deterioration can be attributed to factors such as DNA damage, reduced fertility rate, abnormal mitochondrial structures, oxidative damage, and early oocyte apoptosis. Furthermore, oocytes undergo a time-dependent deterioration process after ovulation when they are not immediately fertilized [17,18]. Delayed fertilization can result in mitochondrial molecular, cellular, and epigenetic abnormalities in oocytes, which are particularly noticeable during assisted reproductive technology procedures when the oocytes are subjected to extended culture before fertilization [19,20,21]. Given these challenges, developing effective strategies to enhance oocyte quality is crucial for improving reproductive outcomes.

Antioxidant treatments may help alleviate oxidative stress and slow the onset of apoptosis during oocyte maturation and development [22,23,24]. A promising avenue for addressing oxidative stress-induced damage in oocytes is the application of natural antioxidants [25,26]. Antioxidants have long been studied for their protective role against oxidative damage [27,28,29]. Among the many well-known antioxidants, tryptophan, a precursor for key molecules such as serotonin and melatonin, is also known to possess antioxidant properties [30,31]. It has been shown to enhance cellular defense mechanisms, reduce oxidative stress, and improve cellular function in various biological contexts. Recent studies have pointed toward its ability to influence the production of serotonin and melatonin, both of which are involved in mitigating oxidative stress [32,33]. These molecules help mitigate oxidative stress by promoting the expression of antioxidant enzymes, such as superoxide dismutase (SOD), and catalase (CAT), which are critical for maintaining cellular integrity [34,35]. Studies have shown that tryptophan treatment can enhance the activity of endogenous antioxidants, offering a potential mechanism by which tryptophan could enhance the antioxidant capacity and quality of oocytes [25,36]. Yet the precise molecular mechanism underlying the beneficial effects of tryptophan on oocyte quality, particularly in aged oocytes, remains underexplored.

To better understand how tryptophan supplementation influences oocyte quality and oxidative status, this study investigates its effects on pig oocytes exposed to H_2_O_2_-induced oxidative stress using a transcriptomic approach. Transcriptomic analysis using RNA-sequencing was employed to capture a comprehensive snapshot of the gene expression changes in response to oxidative stress and tryptophan supplementation. Furthermore, using transcriptomics, this study hopes to provide insights into the molecular mechanism that underpins the effects of tryptophan on oocyte health. These findings would not only shed light on the molecular mechanism that governs oocyte quality under oxidative stress but also pave the way for the development of new strategies aimed at enhancing oocyte quality and reproductive health in livestock.

## 2. Material and Methods

### 2.1. Porcine Oocytes Collection

Fresh ovaries from young sows were harvested from the slaughterhouse and transported to the laboratory in a 0.9% NaCl solution (containing 75 μg/mL penicillin G and 50 μg/mL streptomycin) at 37 °C within 2 h. The ovaries were then washed with the same solution 3 times to remove the blood and other debris. The oocytes from follicles in the range of 3–8 mm were aspirated into a 50 mL centrifuge tube using a 10 mL syringe with a needle. After natural precipitation, the supernatant was discarded, and the oocytes were washed three times with an egg wash solution containing penicillin, streptomycin, NaCl, KCl, NaHCO_3_, CaCl_2_.2H_2_O, KH_2_PO_4_, MgCl_2_.6H_2_O, 4-(2-Hydroxyethyl)-1-piperazineethanesulfonic acid (HEPES), sodium lactate, Na-pyruvate, D-sorbitol, and bovine serum albumen. Cumulus–oocyte complexes (COCs) with more than three layers of cumulus cells and uniform cytoplasm were selected under the microscope. Based on preliminary in vitro dose-dependent experiments evaluating the effect of tryptophan on extrusion rate (Table S1), 50 μM tryptophan demonstrated the optimal activity, and this concentration was selected for subsequent experimental use.

### 2.2. Oocyte Maturation and Treatment with H_2_O_2_ and Tryptophan

In vitro maturation of the oocytes was performed by culturing the COCs in 4-well plates (Nunc, Roskilde, Denmark, 60 mm) with 500 µL of maturation medium per well. The maturation medium employed was TCM199 (Thermofisher Science, 11150059, Waltham, MA, USA). The composition of the culturing medium was porcine epididymal fluid (PEF), cysteine amino acid, and epidermal growth factor (EGF) protein. Approximately 60 COCs were contained in each well, with each well serving as one replicate, and three replicates were used per group. After 44–48 h of culture, metaphase 1 (MI) stage oocytes were obtained. Furthermore, oocytes in H_2_O_2_ and tryptophan groups underwent specific treatments to investigate the effect of oxidative stress and antioxidant supplementation. The oocytes in the H_2_O_2_ and H_2_O_2_+Trp groups were exposed to 100 µM H_2_O_2_ for 0.5 h. The H_2_O_2_+Trp group was further supplemented with 50 μM tryptophan to evaluate the mitigative effect of tryptophan on oxidative stress induced by the H_2_O_2_. The COCs were then cultured in maturation fluid containing tryptophan for 44 h., with conditions carefully controlled at 5% CO_2_, 38.5 °C, and 100% humidity. Throughout this process, the effect of the treatments was carefully monitored by observing and photographing the COCs at different stages of maturation. After culture, the surrounding granulosa cells were digested using 1% hyaluronidase, and the polar body extrusion rate was assessed to evaluate nuclear maturation in vitro. The polar body extrusion rate was determined as follows [37]:

$$PbI\;extrusion\;rate\;\left(\%\right)=\frac{Number\;of\;oocytes\;extruded\;with\;Pb1}{Total\;number\;of\;cultured\;oocyte}\times 100$$

The treatments were crucial to exploring the potential of tryptophan as an antioxidant in protecting oocytes from oxidative stress and improving their quality.

### 2.3. Oxidative Stress Biomarkers Determination

The levels of antioxidant in oocytes were assessed using superoxide dismutase (SOD) kit (No. NJC, A001-3), Malonaldehydes (MDA) kit (NJC, A003-1), Catalase (CAT) kit (NJC, A007-2), and Glutathione peroxidase (GSH-PX) kit (NJC, A005-1), all the kits were acquired from Nanjing Jiancheng bioengineering, Nanjing, China.

### 2.4. RNA Extraction and Assessment of Quality

Transcriptomics analysis of control, H_2_O_2_, and tryptophan-treated oocytes was carried out using a protocol for single-cell RNA-Seq. In brief, two sets of samples were collected from each group (5 oocytes per sample) in lysis buffer. The single-cell collection solution contained cell lysis components and RNAse inhibitors. The nucleic acid sequence with oligo-dT was used for reverse transcription to form cDNA. The cDNAs were amplified by PCR to enrich nucleic acid, and the library was constructed after purification of amplified products, including DNA fragmentation and end repair. The constructed library was sequenced with the lllumina platform, with the PE150 strategy. The sequencing data quality was evaluated using FastQC software (v0.12.0). Following this, reads were processed with Fastp software (v0.20.0) to eliminate short sequences, low complexity reads, and any data of poor quality, along with trimming. The cleaned reads were then aligned to the Sus scrofa reference genome http://www.ensembl.org/Sus_scrofa/Info/Index (accessed on 13 August 2024), and genes with alignment rates between 97.91% and 98.09% were selected for further analysis. The information on the quality assessment of the RNA sequencing is given below in Table 1.

### 2.5. Differential Analysis of Genes

Differentially expressed genes (DEGs) were identified between control vs H_2_O_2_ and H_2_O_2_ +Trp groups using the transcripts per million reads (TPM, Tophat2 v2.1.0) technique. Gene abundance was quantified using RSEM (HTSeq, v 0.5.4 p3). Differential expression analysis was performed with DESeq2 (DEGSeq, v1.12.0). Genes with log2 fold change (log2FC) ≥ 2 and a false discovery rate (FDR) of less than 0.05 were considered to be significantly expressed.

### 2.6. GO Annotation and KEGG Pathway Enrichment Analysis

Gene ontology (GO) (GOSeq, topGO, v1.22) and Kyoto Encyclopedia of Genes and Genomes (KEGG) (KOBAS, v2.0) pathway enrichment analysis were conducted to investigate the potential functions of DEGs. The DEGs were categorized based on GO and KEGG terms. Fisher’s exact test was used to compare the results with genomic background, and significantly enriched GO and KEGG terms were selected for further analysis. To visualize the pathways that were most significantly altered and the genes associated with them, GO tools and Python’s SciPy software were employed using the allwegene website (https://www.majorbio.com/, accessed on 10 September 2024).

### 2.7. Protein–Protein Interaction (PPI) Analysis

Protein–protein interaction (PPI) network analysis was conducted on the DEGs from the comparison of control vs H_2_O_2_ groups and H_2_O_2_ vs H_2_O_2_+Trp groups using the STRING database (https://cn.string-db.org/, accessed on 15 January 2025), with a medium confidence score (0.4). The hub genes within the regulatory network were identified using the CytoHubba plugin in Cytoscape 3.9.3.

### 2.8. Validation of DEGs Quantitative Real-Time PCR (qRT-PCR)

RNA from the blastocyst was extracted using the TRIzol reagent, following the manufacturer’s protocol. Each sample/replicate collected for the RNA extraction contained 40~50 oocytes. The quantity of the RNA was assessed using a Nanodrop spectrometer and gel electrophoresis. RNA was reverse transcribed into complementary DNA (cDNA) using a Master Mix kit, as per the provided guidelines. PCR amplification was performed using the M5-Hiper SYBR Premix EsTaq kit from Bimake (Houston, TX, USA), following the manufacturer’s protocol. The amplification cycle was 30 s for preincubation at 95 °C, denaturation at 95 °C for 95 s, and final melting at 60 °C for 1 s. Data were analyzed using the 2-ΔΔCt method. The primers, provided by Sangon Biotech Co., Ltd., Shanghai, China, are detailed in Table 2.

### 2.9. Statistical Analysis

The results are expressed as the mean ± standard deviation (SD). The analysis was performed using the GraphPad Prism 9.5.1 software. The total number of oocytes included in each group and the number of replicates for each experiment are provided in the figure notes. Comparison between multiple groups was performed using ANOVA with Tukey’s post hoc test.

## 3. Results

### 3.1. Tryptophan Improved the Quality of the Aged Oocytes

**Figure 1:**
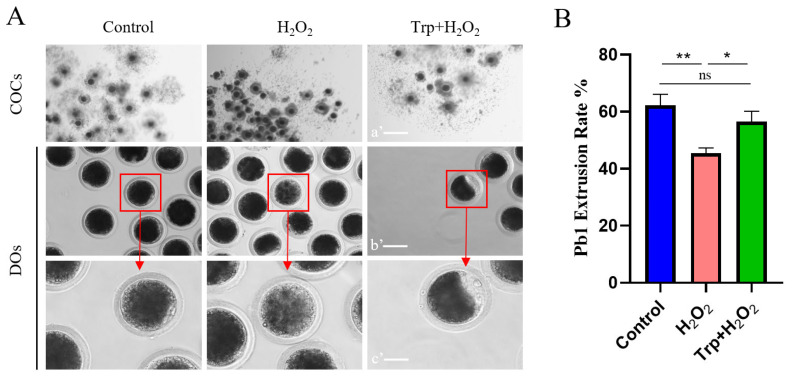
Effect of tryptophan treatment on oocyte maturation and cumulus cell expansion in H_2_O_2_-treated porcine cumulus–oocyte complexes (COCs) (**A**). The polar body extrusion (Pb) in the control, H_2_O_2_-treated, and tryptophan-treated groups (**B**). Results are presented as standard ± SD, n = 3, The *p*-value is represented as * p < 0.05, ** p < 0.01, ns = not significant.

Through the in vitro oocyte maturation test, we explored whether tryptophan could improve the reproductive performance of sows by improving the quality of aged oocytes (Figure 1). In this experiment, the cumulus–oocyte complex was treated with 100 μM H_2_O_2_ oocyte maturation solution for 0.5 h to simulate oocyte senescence. As can be seen from Figure 1A, cumulus cell expansion was good after in vitro culture of normal cumulus–oocyte complexes, while cumulus cell expansion was poor in the H_2_O_2_ group, and the addition of 50 μM tryptophan after H_2_O_2_ treatment could improve this situation. As can be seen in Figure 1B, the polar body (Pb1) extrusion rate % of oocytes in the H_2_O_2_ group were significantly lower than that of the control group (*p* < 0.05), while the addition of tryptophan after H_2_O_2_ treatment restored the oocytes extrusion rate to the level of the control group, indicating a positive effect of tryptophan on oocytes quality.

### 3.2. Tryptophan Supplementation Mitigates the Oxidative Stress

**Figure 2:**
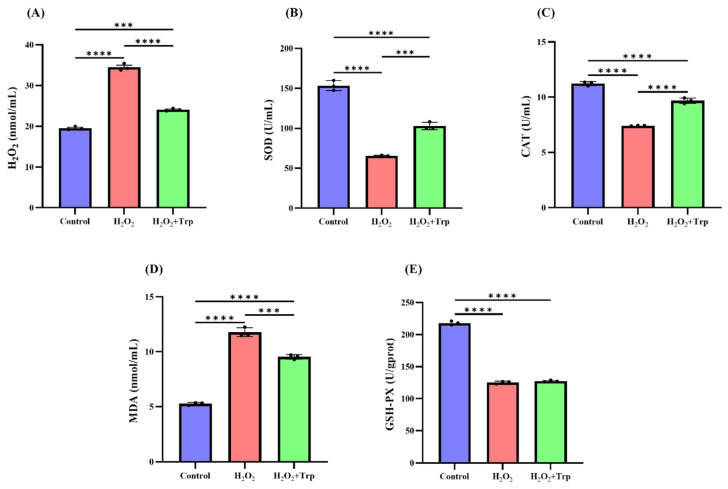
Effect of hydrogen peroxide (H_2_O_2_) and tryptophan on antioxidant biomarkers, including (**A**) H_2_O_2_, (**B**) superoxide dismutase (SOD), (**C**) catalase (CAT) enzyme, (**D**) malonaldehyde (MDA), and (**E**) glutathione peroxide (GSH-PX) in the aged porcine oocytes. Results are presented as standard ± SD, n = 3, The *p*-value is represented as *** p < 0.001, **** p < 0.0001.

Figure 2 shows the results of oxidative stress biomarkers across the three groups, including Control, H_2_O_2_, and H_2_O_2_+Trp. As can be seen in Figure 2A, the H_2_O_2_ concentration significantly increased in the H_2_O_2_ group (34.51 nmol/mL) compared to the control (19.51 nmol/mL) (*p* < 0.0001), reflecting oxidative stress induction. The addition of tryptophan significantly reduced the H_2_O_2_ concentration to 24.07 nmol/mL (*p* < 0.0001), which was closer to the control. SOD activity an antioxidant enzyme that protects against oxidative stress was significantly reduced (65.67 U/mL) in the H_2_O_2_ group compared to the control (153.49 U/mL) (*p* < 0.0001) indicating oxidative stress impairment of antioxidant defense (Figure 2B). The H_2_O_2_+Trp showed significant improvement SOD activity (102.91 U/mL) suggesting partial restoration by tryptophan (*p* < 0.001). As depicted in Figure 2C, the catalase (CAT) activity was significantly reduced in the H_2_O_2_ group (7.42 U/mg prot) compared to the control group (11.23 U/mg prot) (*p* < 0.0001); however, the H_2_O_2_+Trp group had a moderate recovery in catalase activity (9.68 U/mg prot). The MDA levels, a marker of lipid peroxidation, were significantly elevated (*p* < 0.0001) in the H_2_O_2_ group (11.78 nmol/mL)compared to the control group (5.27 nmol/mL), indicating enhanced oxidative damage (Figure 2D). The MDA levels in the H_2_O_2_+Trp group (9.53 nmol/mL) were significantly lower than the H_2_O_2_ group (*p* < 0.0001), still elevated compared to the control, suggesting a partial protective effect of tryptophan. Glutathione peroxidase activity decreased significantly in the H_2_O_2_ group (125.30 U/gprot) compared to the control group (218.12 U/gprot). However, the H_2_O_2_+Trp group showed a slight recovery in GSH activity (127.30 U/gprot), suggesting the protective effect of tryptophan as shown in Figure 2E.

### 3.3. Differentially Expressed Genes (DEGs)

**Figure 3:**
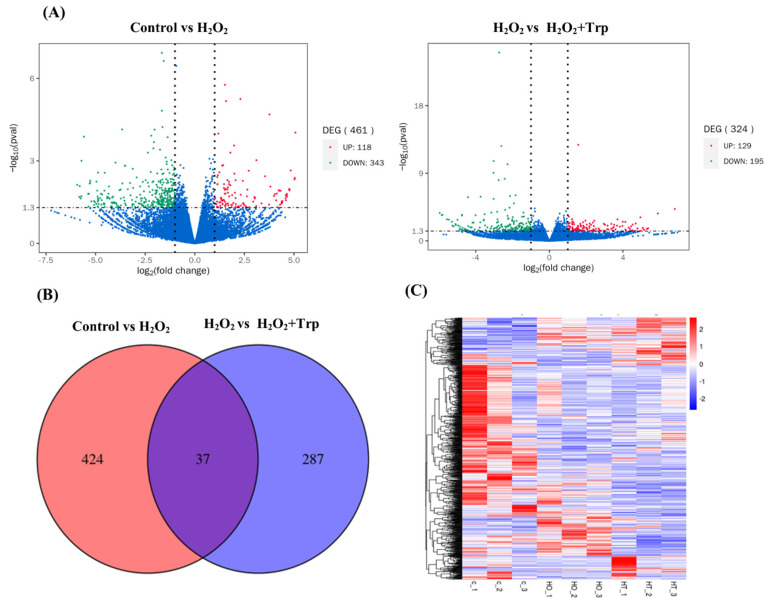
Overview of RNA sequencing results. The volcano plot depicts the RNA-sequencing analysis for control vs H_2_O_2_ and H_2_O_2_ vs H_2_O_2_+Trp groups (**A**). The red dots represent the significantly upregulated genes, and the green dots represent the significantly downregulated genes. The blue dots show non-significant genes. DEGs with a *p* value above 0.05 or with less than 2-fold up- or downregulation are shown in green. The Venn diagram of the DEGs shows the unique and overlapped genes in both the comparison (**B**) and the heatmap illustrating the clustering of the DEGs in groups representing similar samples (**C**).

The gene expression differences between the control and treatment groups were visualized using a volcano plot (Figure 3A), which highlighted the significantly up- and downregulated genes in each comparison. When comparing the control and H_2_O_2_ groups, 118 genes were found to be upregulated, while 343 genes were downregulated, indicating that H_2_O_2_ treatment induced significant changes in gene expression. In the comparison between the H_2_O_2_ and H_2_O_2_+Trp groups, 129 genes were upregulated and 195 genes were downregulated. A Venn diagram further illustrated the overlap and unique sets of DEGs across the three groups (Figure 3B). Among them, 37 genes were common to both comparisons, suggesting that these genes might be crucial in the response to oxidative stress and the mitigation of oxidation by tryptophan treatment. Furthermore, a heatmap-based cluster analysis of the DEGs from each comparison (Figure 3C) showed that genes with similar patterns tended to group, supporting the consistency of the results across biological replicates. The significantly expressed DEGs, along with fold change and *p*-value, are presented in the Supplementary Table S2.

### 3.4. Comparison of GO Enrichment Analysis

**Figure 4:**
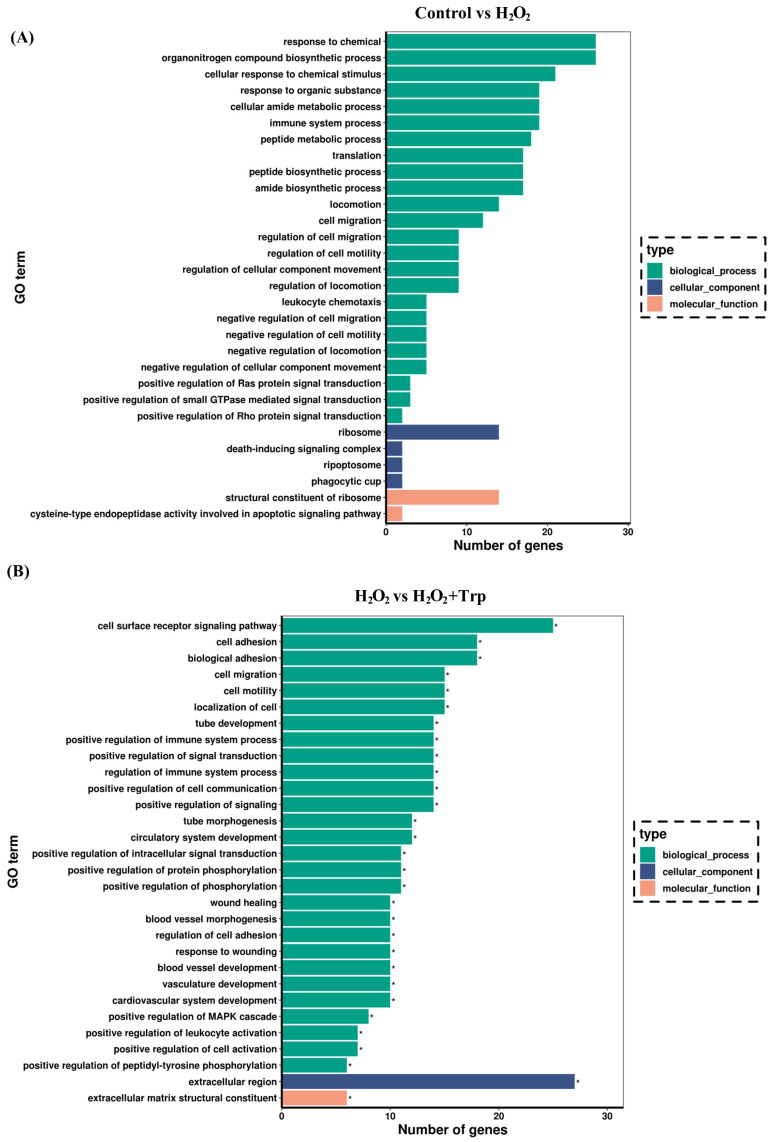
GO annotation of the DEGs in the control vs H_2_O_2_ groups (**A**), and H_2_O_2_ vs H_2_O_2_+Trp groups (**B**). The figure highlights the GO terms that are significantly enriched (* *p* < 0.05). The vertical axis represents the GO categories, which include the biological process (BP), cellular function (CC), and molecular function (MF) categories.

A gene ontology (GO) enrichment analysis was performed on the DEGs identified in the comparison of the control vs H_2_O_2_ group, as well as the H_2_O_2_ vs H_2_O_2_+Trp groups (Figure 4). The DEGs were classified into three main categories, including biological processes (BPs), cellular components (CCs), and molecular functions (MFs), based on their functional annotations. As illustrated in Figure 4A, when comparing the control vs H_2_O_2_ group, enrichment was observed in various BP categories, such as the response to chemicals, biosynthesis of organonitrogen compounds, cellular responses to chemicals, and cellular responses to chemical stimuli. In the MF category, the DEGs were primarily associated with activities involving ribosomes. Figure 4B shows the comparison between the H_2_O_2_ vs H_2_O_2_+Trp groups. In the BP category, the most enriched pathways were cell surface receptor signaling, cell adhesion, and biological adhesion, while in the CC category, the extracellular region was prominent, and in the MF category, the extracellular structural constituent was notably enriched. The relevant data and statistical information are presented in the Supplementary Table S3.

### 3.5. KEGG Enrichment Analysis

**Figure 5:**
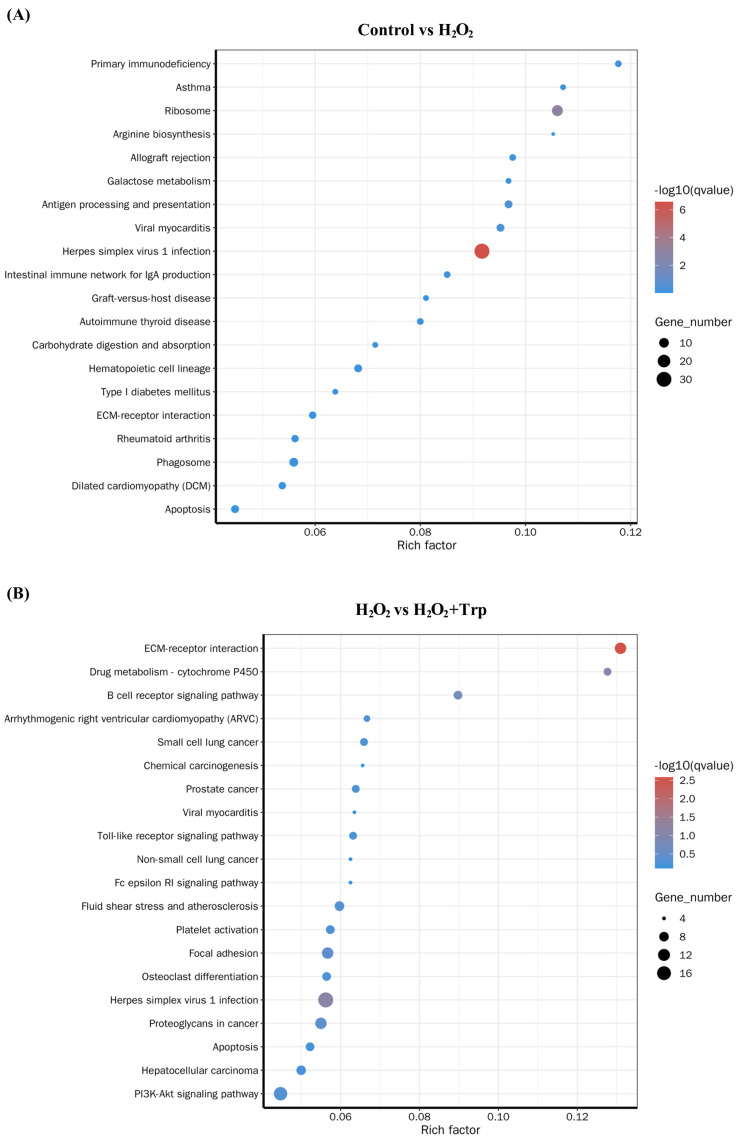
The enriched KEGG pathways analysis of the DEGs in the control vs H_2_O_2_ group (**A**), and the H_2_O_2_ vs H_2_O_2_+Trp groups (**B**). The horizontal axis represents the enrichment factor, and the vertical axis displays the names of the enriched pathways. The size of the dot corresponds to the number of genes linked to each pathway, while the color of the dots indicates the significance level with *p* < 0.05.

A KEGG pathway enrichment analysis was performed on the DEGs from the comparison of the control and H_2_O_2_ groups. The top 20 enriched KEGG pathways for the control vs H_2_O_2_ and H_2_O_2_ vs H_2_O_2_+Trp groups comparison are shown in Figure 5. In the control vs H_2_O_2_ comparison, pathways such as primary immunodeficiency, ribosome, herpes simplex matrix virus 1 infection, antigen processing and presentation, and phagosome were enriched (Figure 5A). These pathways are indicative of a robust immune response triggered by H_2_O_2_-induced oxidative stress. The primary immunodeficiency pathways suggest that H_2_O_2_ exposure may have impaired immune cell function or altered immune response pathways, potentially weakening the host’s ability to respond to oxidative stress. On the other hand, the H_2_O_2_ vs H_2_O_2_+Trp comparison revealed significant enrichment in pathways related to ECM-receptor interaction, Drug metabolism, Focal adhesion, Herpes simplex virus 1 infection, and the P13K-Akt signaling pathway (Figure 5B). The relevant data and statistical information on KEGG pathways are presented in the Supplementary Table S4. The involvement of the P13K-Akt signaling pathway is particularly interesting as it is a well-known pathway associated with cell survival, proliferation, and anti-apoptotic mechanisms [38]. Furthermore, the presence of the drug metabolism pathway in the H_2_O_2_+Trp group suggests that tryptophan may influence metabolic processes, possibly through the modulation of oxidative stress response or by enhancing the metabolism of oxidative by-products. The comparison of these two groups, therefore, highlights the contrasting effects of oxidative stress and the potential mitigating effect of tryptophan supplementation.

### 3.6. Protein–Protein Interaction Network

**Figure 6:**
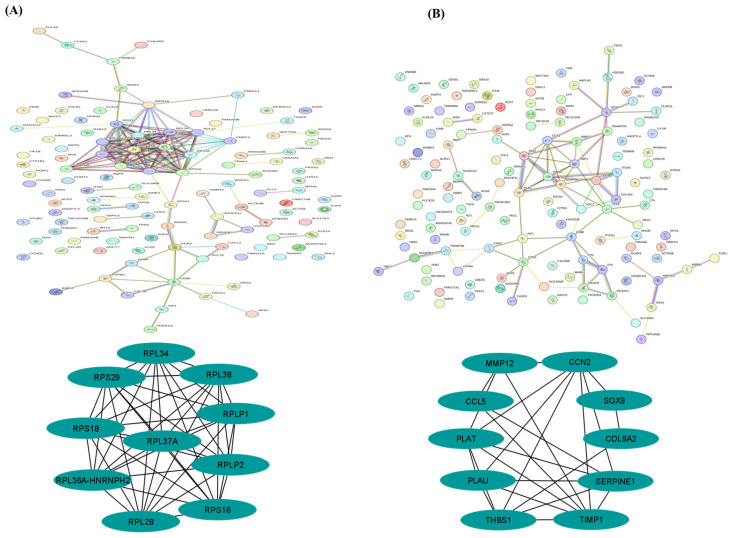
The protein–protein interaction (PPI) network was performed on the significant DEGs in the control vs H_2_O_2_ group and H_2_O_2_ vs H_2_O_2_+Trp groups. The top 10 most important hub genes extracted from the network in the control vs H_2_O_2_ group (**A**), and H_2_O_2_ vs H_2_O_2_+Trp groups (**B**).

The PPI networks were constructed based on the data obtained from the string database (Supplementary file). For the control vs H_2_O_2_ groups comparison, 461 DEGs were selected, while for the H_2_O_2_ vs H_2_O_2_+Trp group comparison, 324 DEGs were submitted to the STRING database to construct the PPI network Figure 6A,B. The top 10 hub genes identified in control vs H_2_O_2_ comparison were *RPLP1*, *RPL34*, *RPL38*, *RPL28*, *RPLP2*, *RPL37A*, *RPL36A-HNRNPH2*, *RPS16*, *RPS18*, and *RPS29* (Figure 6A). These ribosomal proteins are integral to protein synthesis and cellular stress responses, which likely reflect the cellular stress induced by H_2_O_2_ exposure [39]. Their central roles in the network suggest that H_2_O_2_-triggered oxidative stress may disrupt cellular protein synthesis and function, contributing to the cellular dysfunction observed under oxidative stress conditions.

While in the H_2_O_2_ vs H_2_O_2_+Trp comparison the top 10 hub genes were *TIMP1*, *CCN2*, *PLAT*, *THBS1*, *SERPINE1*, *PLAU*, *MMP12*, *COL9A2*, *SOX9*, and *CCL5* (Figure 6B). These genes are involved in extracellular matrix remodeling, cell adhesion, and anti-inflammatory responses, suggesting that tryptophan supplementation may mitigate oxidative stress by promoting tissue repair, reducing inflammation, and enhancing cellular resilience. Notably, the presence of *MMP12*, *COL9A2*, and *SOX9* highlights tryptophan’s potential role in maintaining extracellular matrix integrity and supporting tissue repair processes [40]. Moreover, the important information about these hub genes is reported below in Table 3. The information on string network and statistical analysis is presented in the Supplementary Table S5.

### 3.7. Confirmation of Sequencing Data Through qRT-PCR

**Figure 7:**
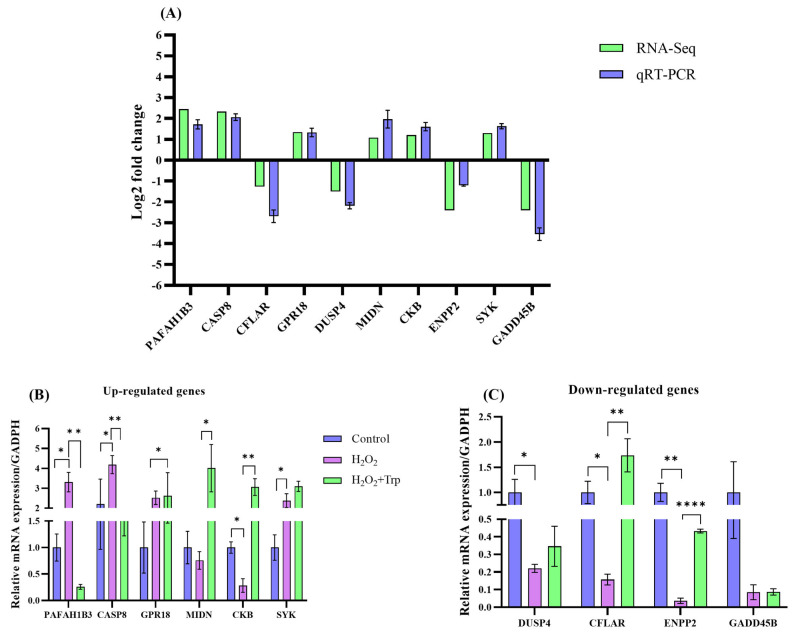
qPCR validation of the related DEGs. (**A**) The comparison of the DEGs and their qRT-PCR. (**B**) The upregulated genes in the control, H_2_O_2_, and H_2_O_2_+Trp groups. (**C**) the downregulated genes among H_2_O_2_, and H_2_O_2_+Trp groups. Gene expression was measured in triplicate for each sample. The expression levels were normalized to *GAPDH* using the 2−∆∆Ct method. The data for the qRT-PCR are represented as mean ± SD. * *p* < 0.05, ** *p* < 0.01, **** *p* < 0.0001.

A total of 10 DEGs were randomly chosen for RNA-sequencing data validation (Figure 7A), among which 6 genes with upregulated (Figure 7B) and 4 genes with downregulated expression (Figure 7C). The qRT-PCR analysis revealed expression patterns that were consistent with those observed in the high-throughput sequencing data. These results confirmed the DEGs expression identified through sequencing. The consistency between the qRT-PCR results and RNA-sequencing data reinforces the reliability and accuracy of the sequencing results. This validation confirms that the identified DEGs truly reflect the biological changes occurring under the experimental conditions, and not due to technical artifacts. Furthermore, these findings emphasize the robustness of the experimental design and the potential significance of these DEGs in understanding the mechanisms underlying the observed effects of oxidative stress and tryptophan supplementation.

## 4. Discussion

Our study utilized an oxidative stress model induced by hydrogen peroxide (H_2_O_2_). The cumulus–oocyte complex (COCs) exposed to H_2_O_2_ exhibited poor cumulus cell expansion and a significant reduction in polar body extrusion rate. The addition of tryptophan improved cumulus cell expansion and enhanced the oocyte environment, which is known to support oocyte developmental potential, to the levels observed in control groups. These results suggest that tryptophan has a protective effect against oxidative stress and can enhance oocyte quality, which is essential for successful fertilization and embryo development. This observation is consistent with previous studies that have shown tryptophan to modulate oxidative stress and cellular function under stressful conditions [41,42]. The increased oxidative stress in oocytes is known to cause cellular damage, especially in the mitochondria and other critical cellular components [43,44]. Tryptophan, an essential amino acid known for its role in protein synthesis and as a precursor for bioactive molecules, has been previously linked to antioxidative properties [45,46]. The results were promising showing that tryptophan treatment effectively reduced the oxidative stress markers, including H_2_O_2_, and restored key antioxidant enzymes activities such as superoxide dismutase (SOD), catalase (CAT), glutathione peroxides (GSH-PX). This suggests that tryptophan not only acts as an antioxidant but also helps maintain the balance of the cellular defense system under stressful conditions. Moreover, tryptophan may exert some of its protective effects indirectly through the synthesis of its derivatives, such as serotonin and melatonin. Both of these molecules have been implicated in regulating oxidative stress and cellular health in various tissues, including oocytes [47,48]. Serotonin, synthesized from tryptophan, has been shown to modulate mitochondrial function and protect cells from oxidative damage [49], while melatonin and serotonin are well known for their potent antioxidant properties and their role in reducing oxidative stress in reproductive tissues [50,51]. Studies have demonstrated that both serotonin and melatonin are present in the ovaries of several mammalian species, where they are involved in regulating oocyte maturation and promoting oocyte survival under oxidative stress conditions [48].

To further elucidate the molecular mechanisms involved in the effects of tryptophan, this study performed RNA-sequencing and assessed gene expression changes between the control, H_2_O_2_, and H_2_O_2_+Trp groups. The gene ontology (GO) enrichment analysis showed the distribution of the differentially expressed genes into various functional categories. In the comparison between the control and H_2_O_2_ groups, the enriched biological processes (BP) included response to chemical, biosynthesis of organonitrogen compounds, and cellular response to chemical stimuli. These pathways are indicative of cellular adaptations to oxidative stress and the initiation of stress response mechanisms [43,52]. In contrast, the H_2_O_2_ vs H_2_O_2_+Trp groups comparison showed enrichment of key pathways related to cell surface receptor signaling, cell adhesion, and biological adhesion. These pathways are involved in maintaining cell integrity and promoting cell–cell interactions [53,54], which may contribute to the restoration of oocyte quality by tryptophan. Furthermore, KEGG pathway enrichment analysis revealed additional insights into the molecular mechanisms underlying the effect of tryptophan. In the comparison between control vs H_2_O_2_ groups, enrichment was observed in pathways related to primary immunodeficiency, ribosome function, and antigen processing and presentation, which are associated with immune responses and protein synthesis. On the other hand, the comparison between the H_2_O_2_ and H_2_O_2_+Trp groups showed enrichment in pathways such as extracellular matrix (ECM)-receptor interaction, focal adhesion, and the P13K-Akt signaling pathway. These pathways are crucial for cell survival, adhesion, and cellular signaling [55,56,57,58], further supporting the role of tryptophan in improving oocyte quality through enhanced cell signaling and structural integrity.

The protein–protein interaction (PPI) network analysis provided additional insights into the key regulatory proteins involved in the response to oxidative stress and tryptophan treatment. The PPI network identified several key genes in both the control vs H_2_O_2_ and H_2_O_2_ vs H_2_O_2_+Trp comparisons. The top hub genes in the control vs H_2_O_2_ comparison were related to ribosomal functions, which are important for protein synthesis and cellular metabolism. In the H_2_O_2_ vs H_2_O_2_+Trp comparison, genes such as *TIMP1*, *CCN2*, and *MMP12* were identified as key players in ECM remodeling and cellular adhesion, which are critical to oocyte quality [59,60,61]. Overall, our study provides strong evidence that tryptophan treatment can improve oocyte quality in aging sows by modulating oxidative stress, enhancing antioxidant defenses, and influencing key gene expression related to cellular signaling and adhesion.

## 5. Conclusions

Our study demonstrated that tryptophan treatments may have improved the quality of aged oocytes by mitigating oxidative stress, restoring antioxidant enzymes, and modulating gene expression involved in cellular responses and adhesion. The molecular mechanisms underlying these effects are complex, involving the regulation of several critical pathways such as the P13K-Akt signaling pathway and ECM-receptor interaction, which are essential for maintaining cellular integrity and functions. These results suggested that tryptophan has significant potential as a therapeutic agent to improve reproductive outcomes in aging sows experiencing age-related oocyte dysfunction. Although the study provides valuable insights, a limitation of the study is the relatively small sample size and the absence of assessment of tryptophan’s effects on in vitro fertilization (IVF) outcomes, such as blastocyst rate. Additionally, the lack of non-tryptophan antioxidant controls and the absence of dose-dependent studies limit the comprehensive understanding of tryptophan’s efficacy. Furthermore, the generalizability of these findings to other species, including humans, remains uncertain. Further research, including in vivo studies and experiments evaluating IVF outcomes, as well as investigations into dose-dependent responses to tryptophan, is necessary to fully understand the long-term effects of tryptophan supplementation on the reproductive system.

## Figures and Tables

**Table 1:** Transcriptome sequencing of the pig oocytes and the statistical analysis of the transcriptome libraries.

Sample	Raw Reads	Raw Bases	Clean Reads	Clean Bases	Error Rate	Q20	Q30	GC Content
Control	82,062,904	12.3 G	81,898,158	12.28 G	0.03%	96.96%	93.15%	51.04%
Control	71,254,586	10.68 G	71,111,626	10.67 G	0.03%	97.36%	93.18%	50.44%
Control	63,043,680	9.45 G	61,790,976	9.27 G	0.03%	94.72%	88.21%	47.34%
H_2_O_2_	91,947,906	13.79 G	90,856,418	13.63 G	0.03%	95.40%	89.33%	48.77%
H_2_O_2_	1.13 × 10^8^	17.02 G	1.13 × 10^8^	16.92 G	0.03%	96.00%	90.36%	48.86%
H_2_O_2_	1.02 × 10^8^	15.27 G	1.01 × 10^8^	15.16 G	0.03%	95.75%	89.98%	48.70%
H_2_O_2_+Trp	95,672,042	14.35 G	94,854,498	14.23 G	0.03%	95.77%	90.01%	48.59%
H_2_O_2_+Trp	1.28 × 10^8^	19.2 G	1.27 × 10^8^	19.11 G	0.03%	96.64%	91.38%	49.43%
H_2_O_2_+Trp	63,072,776	9.46 G	61,947,186	9.29 G	0.03%	95.21%	88.79%	48.36%

**Table 2:** The information on the sequence of the primers for the q-RT-PCR.

Gene	Primer Sequence	Primer Length
*GAPDH*	F: 5′-GAACGGGAAGCTCACTGG-3′ R: 5′-GCCTGCTTCACCACCTTCT-3′	18, 18
*DUSP4*	F: 5′-TGCATCCCAGTGGAAGATAA-3′ R: 5′-GCAGTCCTTCACGGCATC-3′	20, 18
*PAFAH1B3*	F: 5′-CTGGGCTACACACCTGTTTGC-3′ R: 5′-GGAGAGTTTAATGTTGTGGGAAGG-3′	21, 24
*CASP-8*	F: 5′- GTTGTAGCAAGCCGAGATCA-3′ R: 5′-GTGGTCCATGAGTTGGTAGATT-3′	20, 21
*CFLAR*	F: 5′-TGGAGAATGTGGTACGTTAG-3′ R: 5′-AGGAGTGGTGTGGTGGAAG-3′	20, 20
*GPR183*	F: 5’-ACCACCGCTTTGCCTACACGAA-3’ R: 5’-CACCACAGCAATGAAGCGGTCA-3’	22, 22
*MIDN*	F: 5′-CCCCAACTGCCAGGATAGTA-3′ R: 5′-GGT AGTTTTGGGGGTGAGGT-3′	20, 20
*CKB*	F: 5’-ATGCCTGCCCAGAAATGA-3’ R: 5’-GCACTGCCCAGGCAATAA-3’	18, 18
*ENFP2*	F: 5’-GCCCTGATGTCCGTGTATCT-3’ R: 5’-CGTTTGAAGGCAGGGTACAT-3’	20, 20
*GADD45B*	F: 5′-TGACAACGACATCAACATC-3′ R: 5′-GTGACCAGAGACAATGCAG-3′	18, 18
*SYK*	F: 5’ ACTCTGTGGCAGGTATTTCCG-3’ R: 5’ AATAAAGGAAGGCACAGGAGGG-3’	20, 22

**Table 3:** The information on the hub genes from the comparison of the control vs H_2_O_2_ group, and H_2_O_2_ vs H_2_O_2_+Trp group.

Top 10 Hub Genes in Control vs H_2_O_2_ Group Comparison				
Genes id	Genes Description	Genes Regulation	Log2FC	*p*-Value
RPLP1	Ribosomal phosphoprotein1	Down	−0.21973	0.35
RPL34	Ribosomal phosphoprotein34	Down	−0.23755	0.33683
RPL38	Ribosomal phosphoprotein38	Up	0.026498	0.94114
RPL28	Ribosomal phosphoprotein28	Down	−0.32671	0.28
RPLP2	Ribosomal phosphoprotein2	Down	−1.4884	0.01
RPL37A	Ribosomal phosphoprotein37A	Down	−1.1143	0.03
RPL36A-HNRNPH2	Ribosomal phosphoprotein36A- HNRNPH2	down	−0.40596	0.21
RPS16	Ribosomal Protein S16	Down	−1.0723	0.03
RPS18	Ribosomal ProteinS18	Down	−1.208	0.02
RPS29	Ribosomal ProteinS29	Down	−1.0404	0.02
Top 10 hub genes in H_2_O_2_ vs H_2_O_2_+Trp group comparison				
TIMP1	TIMP Metallopeptidase Inhibitor 1	Down	−2.7308	8.45 × 10^−26^
CCN2	Cellular Communication Network Factor 2	Down	−2.7744	0.009
PLAT	Plasminogen activator	Down	−2.2715	6.45 × 10^−11^
THBS1	Thrombospondin 1	Down	−1.1954	0.025
SERPINE1	Serine proteinase inhibitor 1	Down	−2.6116	2.37 × 10^−13^
PLAU	Urokinase plasminogen activator	Down	−1.6891	0.008
MMP12	Matrix Metallopeptidase 12	Up	5.3694	0.024
COL9A2	Type IX collagen	Down	−2.4929	0.031286
SOX9	SRY-Box Transcription Factor 9	Down	−2.1862	0.008
CCL5	C-C Motif Chemokine Ligand 5	Up	1.9127	0.048

## Data Availability

The original contributions presented in this study are included in the article/Supplementary Material. Further inquiries can be directed to the corresponding authors.

## Supplementary Materials

The following supporting information can be downloaded at: https://www.mdpi.com/article/10.3390/biom15070949/s1, Table S1. Preliminary in vitro dose-dependent experiments evaluating the effect of tryptophan on extrusion rate; Table S2. The significantly expressed DEGs in the NC vs H2O2 and H2O2 vs H2O2+Trp; Table S3. The statistical information on GO analysis and the list of enriched genes; Table S4. The relevant data and statistical information on KEGG pathways; Table S5. The information on the string network and the statistical analysis of their interaction.
